# Erratum: A candidate gene-based association study of tocopherol content and composition in rapeseed (*Brassica napus*)

**DOI:** 10.3389/fpls.2013.00069

**Published:** 2013-04-01

**Authors:** Steffi Fritsche, Jinquan Li

**Affiliations:** ^1^Institute for Plant Breeding, Christian-Albrechts-UniversityKiel, Germany; ^2^Quantitative Crop Genetics, Department of Plant Breeding and Genetics, Max Planck Institute for Plant Breeding ResearchCologne, Germany

The publisher regrets that the equation of calculating the percentage of genotypic variation explained by the significant SNPs in the Materials and Methods section was reproduced incorrectly in the above paper.

## Correction

The percentage of genotypic variation explained by the significant SNPs was calculated by $R_{LR}^{2}=1-\text{exp}[-\frac{2}{n}(\log L_{\text{M}}-\log L_{0})]$ where log*L*_*M*_ is the maximum log-likelihood of the model of interest, log*L*_0_ is the maximum log-likelihood of the intercept-only model, *n* is the number of observations (Sun et al., 2010).
